# Toll-Like Receptor 4–Myeloid Differentiation Primary Response Gene 88 Pathway Is Involved in the Shikonin Treatment of CIA by Regulating Treg/Th17 Expression

**DOI:** 10.1155/2018/2428546

**Published:** 2018-12-16

**Authors:** Qiaomei Dai, Ji Li, Yu Yun, Jianwei Wang

**Affiliations:** ^1^Department of Pathology, Heilongjiang University of Chinese Medicine, Harbin, China; ^2^Department of Chinese Formulae, Heilongjiang University of Chinese Medicine, Harbin, China; ^3^Department of Oncology, Traditional Chinese Medical Hospital of Siyang County, Jiangsu, China; ^4^Department of Chinese Medicine, Heilongjiang University of Chinese Medicine, Harbin, China

## Abstract

**Objective:**

To investigate the effect of shikonin on (CIA) collagen-induced arthritis and its influence and mechanism on the balance between Th17 cells and Treg cells.

**Methods:**

Three doses of shikonin were administered orally to mice before the onset of CIA, and celecoxib was used as positive control drug. The arthritis response was monitored visually by macroscopic scoring and hindpaw swelling. Histology of knee was used to assess the occurrence of cartilage destruction and bone erosion. Serum collagen type II (C II) antibody levels associated with CIA were assessed with ELISAs. RT-PCR and quantitative PCR were employed to determine the mRNA expression of cytokines and TLRs in the surface of DCs in the patella with adjacent synovium and spleen in CIA. The expression of cytokines and transcription factors in the peripheral immune organs was tested by Western blotting.

**Results:**

Shikonin treatment suppressed the macroscopic score and incidence of arthritis. Swelling of hind paws, cartilage destruction, and serum anti-C II concentration were delayed with shikonin when compared to controls. Shikonin treatment suppressed the arthritis in a dose-dependent manner. Moreover, the expression of Th17 cytokines (IL-17A) was greatly inhibited both in the synovium and spleen in treated groups compared with those in control groups. The mRNA and protein levels of IL-10 and TGF-*β*, however, were upregulated after shikonin treatment. The expression of Foxp3 in the synovium and spleen was upregulated, and the expression of ROR-*γ*t in the synovium and spleen was downregulated after shikonin treatment through RT-PCR, quantitative PCR, and Western blotting. The DCs in the spleen of shikonin-treated mice had lower expression of TLR4 and MyD88, and the expression of TLR2 and TLR9 in the spleen was not different between the two groups.

**Conclusion:**

Shikonin has anti-inflammatory effects on CIA. Shikonin treatment can inhibit Th17 cytokines expression and induce Treg responses through inhibiting the activation of TLR4/MyD88 pathway.

## 1. Introduction

Synovitis is one of the major pathological changes of rheumatoid arthritis (RA) in autoimmune diseases. Due to the persistence of synovitis, it eventually leads to the damage of cartilage and bone and the dysfunction of joints [1]. The recognized major pathogenesis of RA is immune disorders. CD4^+^T cells and MHC-II antigen presenting cells infiltrate the synovial membrane, initiating a specific immune response, leading to synovitis. As a result, inflammation continues to develop, which can lead to joint swelling, pain, and slow destruction of cartilage and bone, resulting in joint deformity. The roles of T cells in the pathogenesis of RA are not fully understood. However, previous studies reported that Th17 cytokines and CD4^+^CD25^+^FoxP3^+^ regulatory T cells (Tregs) affect each other, and together with cell surface molecules activate synovial macrophages, fibroblast-like cells, and osteoclasts, leading to chronic inflammation and joint destruction [2, 3]. Treg cells is an immunosuppressive CD4 + T cell subsets that secrete IL-10 and TGF-*β*, and play a role in inhibiting T cells and antigen presenting cells, reducing the production of inflammatory cytokines and antibody secretion. Th17 cells are differentiated from Th0 cells secreting IL-17, and IL-17 can interact with TNF-*α*, IL-1, IFN-*γ* to produce synergistic effect, so Th17 is thought to play an important role in mediating inflammatory response. Dendritic cells (DCs) are specialized antigen presenting cells that have the ability to take up, process, and present the antigen information to T lymphocytes. TLR-like receptors are expressed in the surface of DCs and play an important role in the pathogenesis of RA [4].

Zicao, the dried root of Lithospermum erythrorhizon Sieb. et Zucc, Arnebia euchroma (Royle) Johnst, or Arnebia guttata Bunge, is a common herb used in China and other countries. The main chemical component isolated from Zicao is shikonin with a molecular weight of 288 g/mol. Many pharmacological effects were found in shikonin in pharmacological studies. In these roles, the antitumor effect of shikonin was studied most extensively. There is multitarget, multichannel, and multilink performance on the tumor inhibition of shikonin. A variety of tumors have been shown to be inhibited by shikonin in vitro in different ways. Fibroblast-like synovial cells of RA have tumor-like growth characteristics, whose excessive abnormal proliferation and apoptosis play an important role in synovial inflammation and bone destruction of RA [5–8].

The CIA model is widely used to evaluate the role of drugs in RA because of the ability to form aggressive arthritis. In addition to T-cell dysfunction in the CIA mouse model, high titer specific anti-CII antibodies are also produced in animals. Both cell and humoral immunity are involved in the pathogenesis of the CIA. DBA /1(H-2q) mouse strains are the preferred animals for the production of the CIA model, and the resulting CIA model has the destruction of cartilage and bone in common with RA [9–12].

The pharmacological action of shikonin prompted us to use it for the treatment of RA. The immune imbalance of T cells is the main pathogenesis of RA, so we mainly study the immunoregulatory mechanism of shikonin, and the possible roles of Treg, Th17, and TLRs in the pathogenesis of RA in this paper.

## 2. Materials and Methods

### 2.1. Animals

Male DBA/1 Lac/J mice were originally purchased from Slac Laboratories (Shanghai, China). Mice were housed in cages, and water and food were provided ad libitum. DBA/l mice were immunized at the age of 8-10 weeks. This study was reviewed and approved by the Ethics Committee for Experimental Animals of Heilongjiang University of Chinese Medicine, and all animals were treated according to the guidelines of the animal ethical committee.

### 2.2. Materials

Bovine type II collagen was purchased from Chondrex (Washington D.C., USA), and Freund's complete adjuvant (FCA) was purchased from Sigma (St. Louis, MO). Shikonin was purchased from Chengdu Must Bio-Technology Co., Ltd. (Chengdu, China). ELISAs used kits for Goat Anti-Mouse Collagen Type II from BD Biosciences (New York, USA). TRIzol reagent was purchased from Invitrogen Corporation (California, USA), and Primers were purchased from Sangon Biotech Co., Ltd. (Shanghai, China). RNA PCR Kit (AMV, Ver. 3.0 and EX TAQ R-PCR Version 2.1) were obtained from TaKaRa Biotechnology Co., Ltd. (Dalian, China). Antibodies against Foxp3 was purchased from Cloud-Clone Corp., (California, USA), and Rabbit anti IL-17 was purchased from Beijing Biosynthesis Biotechnology Co., Ltd. (Beijing, China)

### 2.3. Induction of CIA

Bovine type II collagen was emulsified in an equal volume of FCA. On day 1, the mice were immunized intradermally at the bottom of the tail with 100*μ*l of emulsion (100*μ*g of collagen). On day 21, the animals were given booster injections intraperitoneally with 100*μ*g of Bovine type II collagen [2, 13].

### 2.4. Treatment Protocol of Shikonin in CIA Arthritis

DBA/1 Lac/J mice without signs of arthritis on day 21 were divided into five groups with ten mice in each group randomly, which is respectively for negative control group, low dose group of shikonin (2mg/kg), medium dose group of shikonin (3.5mg/kg), high dose group of shikonin (5mg/kg), and celecoxib group (30mg/kg). Shikonin and celecoxib were given orally to mouse once every two days for 60 days, and PBS without Collagen II were given to negative control group.

### 2.5. Assessment of CIA

The arthritis score was evaluated from the 21st day of immunization. Mice were considered to have arthritis when their paws, ankles, and knees were significantly red or swollen. The arthritis scoring criteria were as follows: 0-no changes; 0.25-1 to 2 toes red or swollen; 0.5-3 to 5 toes red or swollen; 0.5-swollen ankle; 0.5-swollen footpad; 0.5-severe swelling and ankylosis. Mice were scored every other day for erythema and swelling by two independent observers [2, 13]. Micrometer measurements of foot depth were performed at regular intervals using micrometer caliper [14].

### 2.6. Histology

Mice were sacrificed by ether anesthesia. The knee joint of the immobilized CIA mouse in 4% formalin was placed in the embedding box, and alcohol was used as dehydrating agent. After dehydration, paraffin embedding, decalcification, slicing, and patch were used for hematoxylin and eosin staining. Hematoxylin and eosin staining was used to assess the degree of inflammation (0-3) and the degree of cartilage destruction (0-4) and bone infiltration (0-3), and the histopathological progression of each group of mice was evaluated [13, 15].

### 2.7. ELISAs

Levels of Collagen Type II in blood of CIA were measured using ELISA kits, in accordance with the manufacturers' instructions.

### 2.8. Isolation of Spleen CD11c+DCs

Mice were sacrificed after anesthesia. The collected spleens were placed on a 200 mesh stainless steel screen; cold PBS was added and carefully grounded with a 1 ml syringe plunger. The screen was repeatedly washed with cold PBS, and the cell suspension was collected in a 10 ml centrifuge tube. We discarded the supernatant and added ELS lysate to the cell pellet in a ratio of 1:3 to 5 and gently pipetted. Centrifuged at 1000 rpm for 5 min, the supernatant was discarded and the pellet was collected. It centrifuged 3 times with Hank's solution and added 1 ml of PBS. The cells were suspended and counted using a cell counting plate. CD11c+ cells were isolated with the CD11c MicroBeads according to the manufacturer's instructions [16].

### 2.9. Polymerase Chain Reaction (PCR) Amplification

After CIA mice were sacrificed, the synovial tissue near the patella of the CIA mouse was collected to extract the total RNA for reverse transcription PCR (RT-PCR) and quantitative PCR. The CD11c+ cell suspension was counted and RNA was extracted, and the expression of TLR receptors was determined by quantitative RT-PCR [17]. Primer sequences and conditions for PCR were from the paper described previously [7]. Primer sequences for PCR were as follows: *β*-actin, forward 5'- AGC GGT TCC GAT GCC CT - 3', reverse 5'- AGA GGT CTT TAC GGA TGT CAA CG- 3', TGF-*β*, forward 5'- AAA CGG AAG CGCATC GAA 3', reverse 5'- GGG ACT GGC GAG CCT TAG TT 3', Foxp3, forward 5'- GGC CCT TCT CCA GGA CAG A, reverse 5'- GCT GAT CAT GGC TGG GTT GT-3', ROR-*γ*t reverse 5'-TGT TTT ATG GGG TTT GGG TAT G-3', reverse 5'-CTG TGT GGA TGT GTG TCT CTG ATT A-3', Interleukin-17A, forward 5'-AGT GAA GGC AGC AGC GAT CAT-3', reverse 5'-CGC CAA GGG AGT TAA AG-3'. TLR-2, forward 5'-GCA AAC GCT GTT CTG CTC AG-3' reverse 5'-AGG CGT CTC CCT CTA TTG TAT T-3'. TLR-4, forward 5'-ATG GCA TGG CTT ACA CCA CC-3', reverse 5'-GAG GCC AAT TTT GTC TCC ACA-3', TLR-9, forward 5'-ATG GTT CTC CGT CGA AGG ACT-3', reverse 5'-GAG GCT TCA GCT CAC AGG G-3', MyD88, forward 5' TCA TGT TCT CCA TAC CCT TGGT-3', reverse 5'-AAA CTG CGA GTG GGG TCA G-3'. Transcripts were quantified using the EX TAQ R-PCR. The PCR was initiated for 2 min at 95°C, continued with 40 cycles of 10 sec at 95°C and 40 sec at 60°C. The fold change in expression of each gene was calculated using the ΔΔCt method, with the housekeeping gene *β*-actin mRNA as an internal control [17].

### 2.10. Western Blot Analysis

Total proteins were extracted from the dissected spleen. The spleen was solubilized in the cytoplasmic lysis buffer with a protease inhibitor and the nuclei were lysed in the nuclear lysis buffer as described previously. After the protein concentration was detected, the gel and the electrophoretic transfer membrane were sealed and incubated with the primary and secondary antibodies. Bands were detected using a SuperSignal west chemiluminescence substrates and visualized with a laser-4000 [18].

### 2.11. Statistical Analysis

Data were presented as the means ± SDs. The statistical significance of differences was analyzed by Student's t test and one-way analysis of variance (ANOVA), unless stated otherwise. Differences with* p* values below 0.05 were judged to be significant.

## 3. Results

### 3.1. Anti-Inflammatory Role of Shikonin on CIA

**Figure 1:**
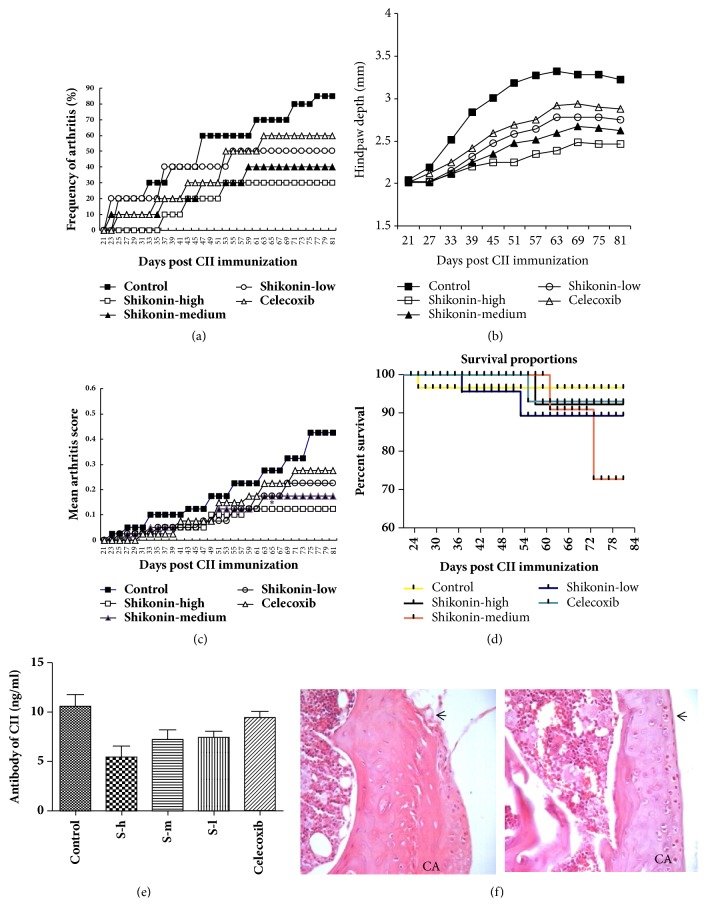
(a) Frequency of arthritis. (c) Mean arthritis macroscopic score. (d) Survival proportions of CIA mice. Immunized DBA/1 mice without signs of CIA were selected and divided into five separate groups. Mice were treated with shikonin (three doses), celecoxib, and with 0.1ml PBS as control group for 60 days. Clinical arthritis score and incidence were monitored throughout the experiment. The data represent macroscopic score and mean incidence of arthritis in every group. *∗*indicates* P*<0.05 between treated and control groups. (b) Hindpaw thickness of every group. The effect of shikonin on the clinical progression of developing CIA as monitored by foot swelling. Measurements of the hindpaw depth were performed every six days. (e) The level of anti-CII IgG antibody in serum. Levels of Collagen Type II in blood of CIA were measured using ELISA kits. Note: S-h: high dose of shikonin, S-m: medium dose of shikonin, and S-l: low dose of shikonin. (f) HE staining of the knee joint. Sections of the knee were collected on day 81 and stained with H&E stain (40X). Left: PBS-treated mice with Collagen II induced arthritis, showing severe cartilage surface disruption, infiltrate of inflammatory cells, and bone erosion (arrows). Right: Mice with CIA treated with 5mg/kg shikonin, showing marked improvements with intact cartilage surface (arrows). Note: CA: cartilage.

After secondary immunization, redness or swelling was gradually found on the joints of CIA mice. CIA mice without arthritis symptoms were selected on day 21 and administered with shikonin and with celecoxib as positive control drug. After shikonin treatment, arthritis symptoms were relieved, and the arthritis score and arthritis incidence were significantly lower than those of the control group (Figures 1(a) and 1(c)). Survival proportions of CIA mice (Figure 1(d)). Joint thickness (paw swelling) was delayed after shikonin treatment (Figure 1(b)). Serum antitype II collagen antibody levels in high dose group of shikonin, medium dose group of shikonin, and low dose group of shikonin were significantly reduced compared with control group (Figure 1(e)) (Table 1).

Pronounced amelioration of the influx of inflammatory cells, cartilage destruction, and bone erosion was found when the shikonin-treated animals were compared with the vehicle-treated animals (Figure 1(f)) (Table 2)

### 3.2. Expression of Cytokines and Transcription Factors in the Arthritic Joint and Spleen

**Figure 2:**
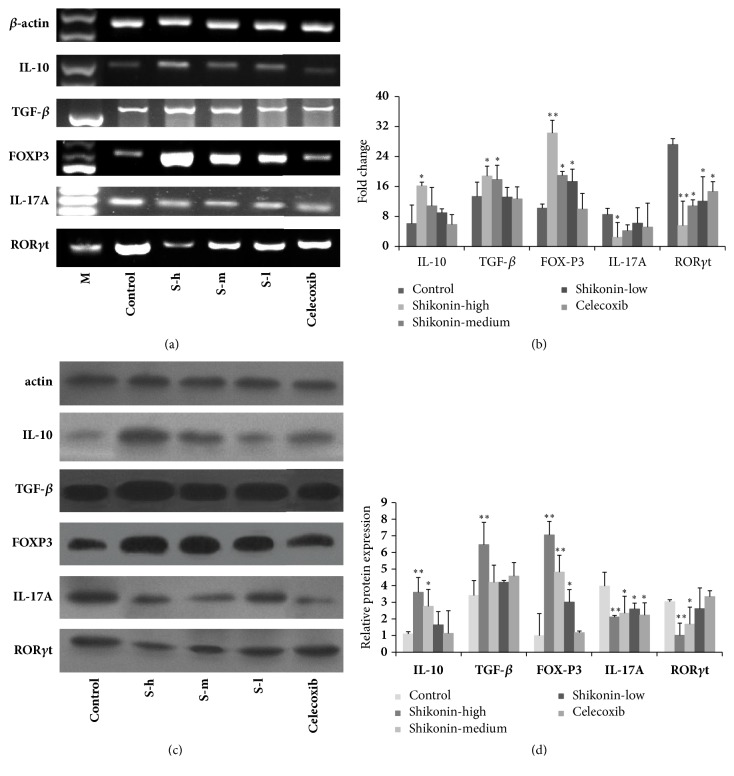
(a) Expression of cytokine and transcription factor in the arthritic joint. The expression of IL-17A, ROR-*γ*t, IL-10, TGF-*β*, and Fox-P3 was determined by using RT-PCR analysis. *β*-actin was used for loading control. Note: S-h: high dose of shikonin, S-m: medium dose of shikonin, S-l: low dose of shikonin. (b) The density histogram data of cytokine and transcription factor in the arthritic joint. The expression of IL-17A, ROR-*γ*t, IL-10, TGF-*β*, and Fox-P3 was determined by using quantitative RT-PCR analysis. *β*-actin was used for loading control. The density histogram data were from three-separated quantitative RT-PCR analysis (mean±SE), which represents the relative expression of IL-17A, ROR-*γ*t, IL-10, TGF-*β*, and Fox-P3, respectively. (c) The protein expression in the spleen. The expression of IL-17A, ROR-*γ*t, IL-10, TGF-*β*, and Fox-P3 was analyzed by Western blot as described in Materials and Methods. *β*-actin was used for loading control. (d) The density histogram data from three-separated Western blot analysis (mean±SE), which represents the relative expression of IL-17A, ROR-*γ*t, IL-10, TGF-*β*, and Fox-P3. *∗* indicates* P*<0.05 or *∗∗* indicates* P*<0.01 between treated and control groups.

To elucidate the changes of Th17/Treg after the treatment of shikonin, the expression of mRNA and protein of cytokines and transcription factors in the arthritic joint and peripheral immune organs (spleen) in CIA were detected. The mRNA expression of cytokines and transcription factors were determined by RT-PCR (Figure 2(a)) and quantitative RT-PCR (Figure 2(b)) as shown in Figure 2. Marked decreases in IL-17A and ROR-*γ*t mRNA expression were detected in shikonin-treated mice compared with that of control group. Enhanced mRNA levels for IL-10 and TGF-*β* were more impressive. Interestingly, Foxp3 expression was upregulated after shikonin treatment. Protein expression of cytokines and transcription factor in peripheral immune organs (spleen) were determined by Western blot. Enhanced protein levels for IL-10, TGF-*β*, and Foxp3 were more impressive. Marked decreases in IL-17A and ROR-*γ*t protein expression were detected in shikonin-treated mice compared with those of control group (Figures 2(c) and 2(d)).

### 3.3. The mRNA Expression of Surface Signaling Pathways Molecules of DCs

**Figure 3:**
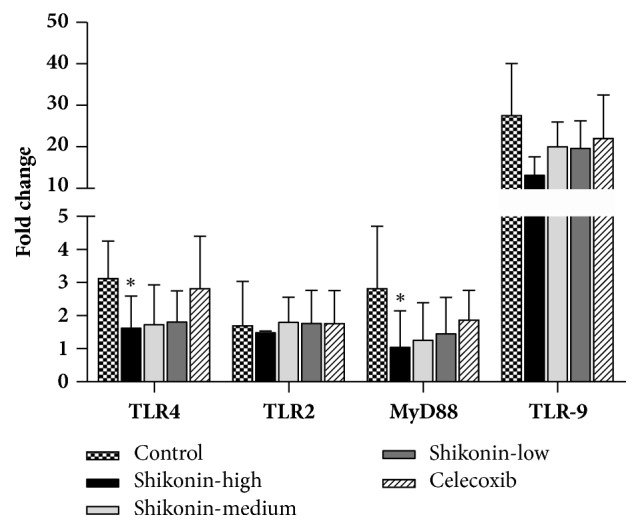
Effect of shikonin on expression of TLRs in the DCs. Representative results show changes in the expression of TLR2, TLR4, Myd88, and TLR9 in spleen DCs, determined by real-time PCR analysis at 81 days after immunization. *∗*,* p* < 0.05* vs.* control, *∗∗ p* < 0.01* vs.* control.

To elucidate the mechanism of the treatment, we investigated the expression of toll-like receptors in the DCs. The expression levels of TLR4 and MyD88 in the spleen DCs of shikonin group were significantly lower than those in the spleen DCs of control group, while the expression of TLR2 and TLR9 was not significantly different (Figure 3).

## 4. Discussion

CIA is corrosive arthritis, with lesions similar to human RA [18–22], and it can cause deformity and dysfunction on mouse and rat. Anticollagen antibodies often occur in patients of RA which may also appear on collagen-induced arthritis model [23, 24]. In order to study the effect of shikonin, three doses of shikonin were given to CIA mice and with celecoxib as a control drug. Mouse body weight of high dose and middle dose group of shikonin showed decline compared with that body weight before modeling (no statistical significance). However, a significant improvement in the arthritis symptoms in the CIA mice with high doses of shikonin was confirmed by histology. The results confirmed that each treated group was able to reduce the degree of joint swelling and arthritis score, and high-dose shikonin group was better than other treated groups. Shikonin treatment suppressed the arthritis in a dose-dependent manner.

Type II collagen is main component of protein of articular cartilage, which forms the network structure of fibers, together with hyaline cartilage and proteoglycan constituting a very strong structure to support the pressure. The number of positive anti-antibody of C II, RF-positive and erythrocyte sedimentation rate of the average levels in patients with RA were greater than those of anti-C II antibody-negative group of RA, suggesting a certain relationship between anti-C II antibodies and disease progression and severity of RA [25–27]. Shikonin is known as having anti-inflammatory effect [28, 29]. It was confirmed that shikonin-treated group and celecoxib-treated group can reduce serum anti-C II concentration of CIA mouse, whereas statistical significance was found in shikonin-treated group.

A large number of T cells were found in the synovial membrane of RA. Treg cells play an active role in preventing the spontaneous development of systemic autoimmunity, and previous research has focused on whether deficiencies in Treg cells activity might contribute to the development of autoimmune diseases such as RA [30]. Treg cells are present in the synovial fluid of RA patients and are potent suppressors of responder T-cell proliferation and TNF and IFN-*γ* production; in addition, activated T cells in the synovium also seemed to be suppressed by Treg cells [1, 31]. On the other hand, IL-17 is a key driver of inflammation, and it is present in the rheumatoid synovium of the joints of arthritic mice [32, 33]. Mice deficient in IL-17 have reduced severity of arthritis, and those with increased IL-17 level have exacerbated disease [34, 35]. Inflammatory cytokines are found selectively to be recruited to the synovial cavity in rheumatoid arthritis, so various related cytokines in the synovial tissue were detected in this paper [36, 37]. Shikonin group significantly reduced the expression of IL-17A in synovium compared with the negative control group, whereas IL-10 and TGF-*β* expression were found to increase compared with that model group. Transcription factors of Th17 and Treg can bind to each other and inhibit the function of each other at the molecular level [32, 33], so ROR-*γ*t and Foxp3 were amplified in this research. There were a marked decrease in ROR-*γ*t expression and a marked increase in Foxp3 expression compared with control groups in inflamed synovium and patellar cartilage of shikonin-treated group, which showed shikonin exert anti-inflammatory effects through inhibition of Th17 cells and induction of Treg cells.

T cells patrol the body except the brain and other immune cells reach the site of tissues and organs, which interact with that carrying specific antigens recognized by the APC peptide. T cells are then amplified and start the immune response [38–40]. To further understand the mechanism of action of Treg cell and Th17, protein of related cytokines and transcription factors in spleen in CIA were detected. The results showed that the expression of cytokines and transcription factors in the spleen were consistent with those in the synovium.

TLR9 is expressed in dendritic cells and can recognize the unmethylated CpG motif and develop Th1 immune responses in major-infected susceptible BALB/c mice [41]. DC cells can be activated by TLR2-6 microbe stimulating signals, and in this process, Treg can also be induced to secrete TGF-*β* and IL-10 [42]. Several reports have demonstrated that TLR4 signaling is needed for the activation and maturation of dendritic cells, which acquire the competent phenotype to preferentially differentiate naïve T cells to the Th1 or Th17 pattern. TLR4 ligation is important for the activation of Th1 or Th17 responses, while TLR4-deficiency can lead to increased expansion of CD4+ CD25+ regulatory T cells. For example, activation of TLR4 expressed in T cells promotes suppressive function of regulatory T cells and Th17 cells are considered divergent and mutually inhibitory [43]. VGX-1027 and TAK-242 are inhibitors of TLR4. The development of immunoinflammatory and autoimmune diseases in different animal models such as pleurisy, rheumatoid arthritis [44], type 1 diabetes mellitus [45], systemic lupus erythematosus [46], and inflammatory bowel diseases [47] can be inhibited by VGX-1027. TAK-242 dampens the development of immunoinflammatory and autoimmune diseases in different animal models such as an amyotrophic lateral sclerosis, experimental autoimmune myositis, and deficiency of IL-36Ra [48–50]. Our results showed that the DCs in the spleen of shikonin-treated mice had lower expression of TLR4 and Myeloid differentiation primary response gene 88 (MyD88). MyD88, as a cytosolic adaptor molecules, is one of transmits signals of TLR4 [49]. Shikonin may suppress CIA by regulating Treg/Th17 expression through inhibiting the activation of TLR4/MyD88 pathway.

All applicable international, national, and/or institutional guidelines for the care and use of animals were followed.

## Figures and Tables

**Table 1:** Antibody of CII in serum ($\overset{-}{x}\pm s$, *n* = 5).

Group	CII(ng/ml)
Control	10.59±2.20
Shikonin-high	5.27±2.00*∗∗*
Shikonin- medium	7.02±2.24*∗*
Shikonin-low	7.42±1.28*∗*
Celecoxib	9.45±1.20

Note: ^∗^*P *<0.05,^ ∗∗^*P *<0.01 versus controls.

**Table 2:** Histology of knee joints after shikonin treatment ($\overset{-}{x}\pm s$, *n*=5).

Group	Infiltrate	Cartilage destruction	Bone erosion
Control	1.20±0.5	1.10±0.30	1.90±0.70
Shikonin-high	0.71±0.4*∗∗*	0.19±0.28*∗∗*	0.57±0.37*∗∗*
Shikonin- medium	0.83±0.4*∗*	0.31±0.28*∗∗*	0.78±0.43*∗∗*
Shikonin-low	1.10±0.5	0.79±0.40*∗*	1.13±0.53*∗*
Celecoxib	0.80±0.5*∗*	0.93±0.40	1.72±0.36

Note: ^∗^*P *<0.05, ^∗∗^*P *<0.01 versus controls.

## Data Availability

All data included in this study are available upon request by contact with the corresponding author.
