# Corrigendum to “Toll-Like Receptor 4–Myeloid Differentiation Primary Response Gene 88 Pathway Is Involved in the Shikonin Treatment of CIA by Regulating Treg/Th17 Expression”

**DOI:** 10.1155/2019/2198708

**Published:** 2019-03-07

**Authors:** Qiaomei Dai, Ji Li, Yu Yun, Jianwei Wang

**Affiliations:** ^1^Department of Pathology, Heilongjiang University of Chinese Medicine, Harbin, China; ^2^Department of Chinese Formulae, Heilongjiang University of Chinese Medicine, Harbin, China; ^3^Department of Oncology, Traditional Chinese Medical Hospital of Siyang County, Jiangsu, China; ^4^Department of Chinese Medicine, Heilongjiang University of Chinese Medicine, Harbin, China

In the article titled “Toll-Like Receptor 4–Myeloid Differentiation Primary Response Gene 88 Pathway Is Involved in the Shikonin Treatment of CIA by Regulating Treg/Th17 Expression” [1], reference [4], J. A. Carlsson et al. [2], was incorrectly included in the reference list and should be replaced with M. F. Roelofs et al. [3].
